# Genetic differentiation and connectivity of morphological types of the broadcast‐spawning coral *Galaxea fascicularis* in the Nansei Islands, Japan

**DOI:** 10.1002/ece3.1981

**Published:** 2016-02-03

**Authors:** Yuichi Nakajima, Yuna Zayasu, Chuya Shinzato, Noriyuki Satoh, Satoshi Mitarai

**Affiliations:** ^1^ Marine Biophysics UnitOkinawa Institute of Science and Technology Graduate University1919‐1 TanchaOnnaOkinawa904‐0495Japan; ^2^ Marine Genomics UnitOkinawa Institute of Science and Technology Graduate University1919‐1 TanchaOnnaOkinawa904‐0495Japan

**Keywords:** Conservation, cryptic species, genetic diversity, microsatellite, population genetics

## Abstract

Population connectivity resulting from larval dispersal is essential for the maintenance or recovery of populations in marine ecosystems, including coral reefs. Studies of species diversity and genetic connectivity within species are essential for the conservation of corals and coral reef ecosystems. We analyzed mitochondrial DNA sequence types and microsatellite genotypes of the broadcast‐spawning coral, *Galaxea fascicularis*, from four regions in the subtropical Nansei Islands in the northwestern Pacific Ocean. Two types (soft and hard types) of nematocyst morphology are known in *G. fascicularis* and are significantly correlated with the length of a mitochondrial DNA noncoding sequence (soft type: mt‐L; hard type: mt‐S type). Using microsatellites, significant genetic differentiation was detected between the mitochondrial DNA sequence types in all regions. We also found a third genetic cluster (mt‐L+), and this unexpected type may be a cryptic species of *Galaxea*. High clonal diversity was detected in both mt‐L and mt‐S types. Significant genetic differentiation, which was found among regions within a given type (*F*_ST_ = 0.009–0.024, all *P*s ≤ 0.005 in mt‐L; 0.009–0.032, all *P*s ≤ 0.01 in mt‐S), may result from the shorter larval development than in other broadcast‐spawning corals, such as the genus *Acropora*. Nevertheless, intraspecific genetic diversity and connectivity have been maintained, and with both sexual and asexual reproduction, this species appears to have a potential for the recovery of populations after disturbance.

## Introduction

Scleractinian reef‐building corals constitute the framework of reef ecosystems and are especially endangered due to a multitude of rapid, oceanic environmental changes caused by anthropogenic disturbances, including extreme thermal stress from increasing sea surface temperatures (e.g., Hoegh‐Guldberg 1999; Cantin et al. 2010), ocean acidification from increasing atmospheric CO_2_ (e.g., Hoegh‐Guldberg et al. 2007), and various local problems such as sediment pollution or nutrient influx from runoff (e.g., McCulloch et al. 2003). Population connectivity resulting from larval dispersal is essential for the maintenance or recovery of populations in marine ecosystems. Fertilized larvae of the broadcast‐spawning corals disperse after spawning, and dispersal distances are dependent upon larval characteristics, such as the duration of the larval period, or time until settling (e.g., Nishikawa et al. 2003; Ayre and Hughes 2004). Therefore, broadcast‐spawning corals are likely to maintain high genetic connectivity among populations. While various studies have reported high genetic connectivity in broadcast‐spawning taxa such as the genus *Acropora* (e.g., Nishikawa et al. 2003; Ayre and Hughes 2004; Nishikawa and Sakai 2005a; Underwood et al. 2009; Nakajima et al. 2010, 2012a; Davies et al. 2015), some broadcast‐spawning species have lower genetic connectivity than expected, due to the short duration of larval dispersal (e.g., *Platygyra daedalea*: Miller and Ayre 2008). Population genetic analysis in another genus will provide further insights into the genetic connectivity of the broadcast‐spawning corals in this region. We conducted population genetics of the broadcast‐spawning coral, *Galaxea fascicularis* (Linnaeus, 1767). This is the first report of a population genetic study of the genus *Galaxea* focusing on a large geographic range in an island reef system.

**Figure 1:**
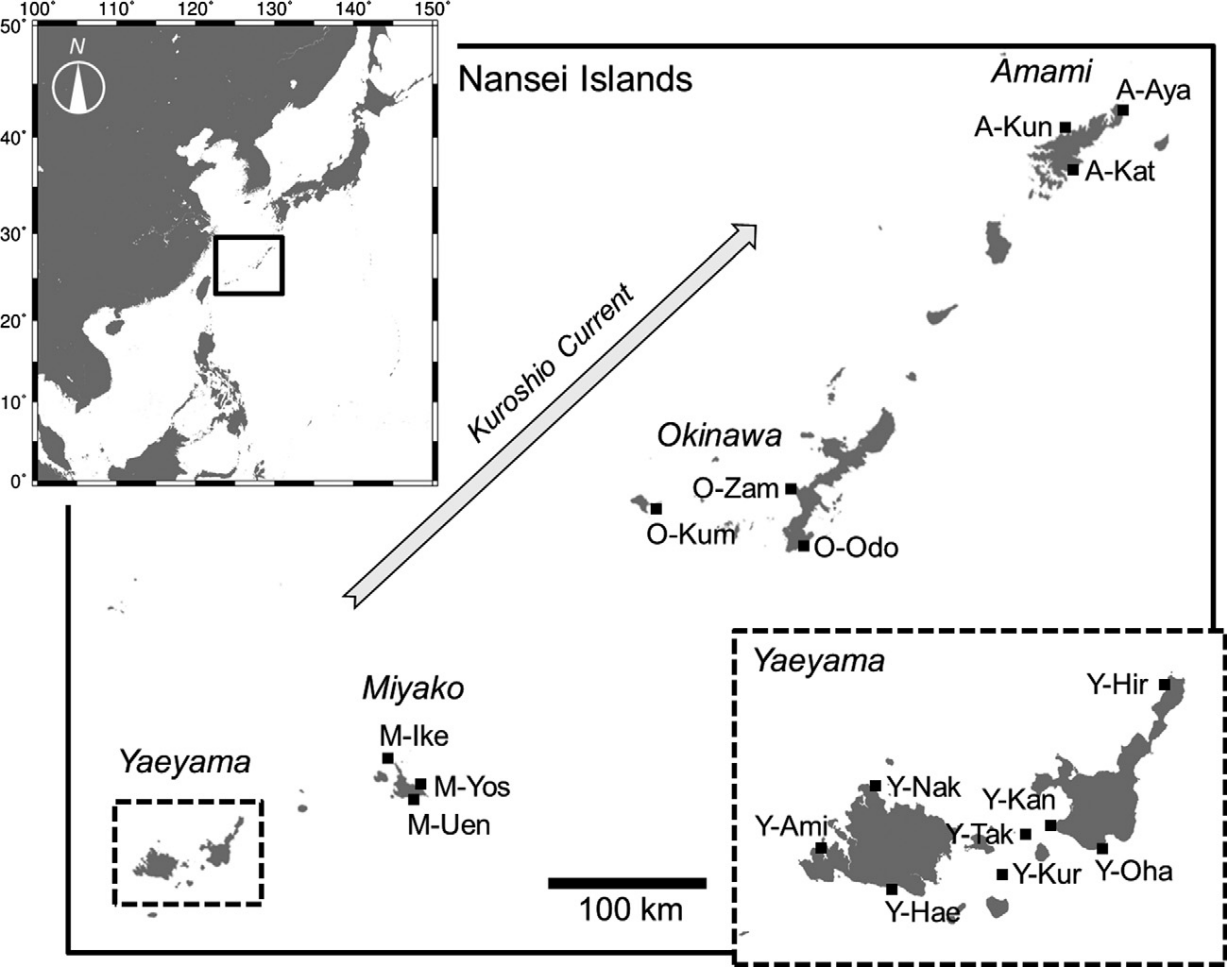
Collection sites for *Galaxea fascicularis* in the Nansei Islands in southwestern Japan.

*Galaxea fascicularis* is mainly distributed in reef areas in the Indo‐western Pacific region (Veron 2000). It is a gonochoric, broadcast‐spawning species; female colonies produce egg bundles, and male colonies form bundles consisting of sperm and infertile pseudo‐eggs (e.g., Harrison 1988; Hayakawa et al. 2007; Keshavmurthy et al. 2012). This species is classified into soft and hard types based on nematocyst morphology (Hidaka 1992), and this morphological characteristic is correlated with the length of the noncoding region between the mitochondrial genes *cyt* b and *nad* 2 (mt‐Long: soft type; mt‐Short: hard type) (Watanabe et al. 2005; Abe et al. 2008a). Significant genetic differentiation between these mt‐Long (mt‐L) and mt‐Short (mt‐S) types was shown at Zampa, on Okinawa Island using polymorphic nuclear microsatellite markers (Nakajima et al. 2015). However, significant genetic differentiation between mt‐L and mt‐S has not been demonstrated in other regions. Studies of boundaries between coral species are needed to estimate species diversity, which is essential for coral conservation. This species is one of the dominant, easily identified species at some reef sites in the Nansei Islands located in southwestern Japan. These islands are subtropical in nature, and a strong current, the Kuroshio Current, flows from southwest to northeast along the islands (Fig. 1). This current is considered a major factor in the expansion and maintenance of coral reefs and reef‐dwelling organisms in the Nansei Islands (Nishihira and Veron 1995; Nishihira 2004).

In broadcast‐spawning corals in this region, the genetic connectivity of *Acropora digitifera* (Nishikawa and Sakai 2005a; Nakajima et al. 2010), *Acropora tenuis* (Nishikawa et al. 2003), and *Coelastrea aspera* (formerly *Goniastrea aspera*) (Nishikawa and Sakai 2003, 2005b) has been analyzed using allozyme or microsatellite markers. The genetic differentiation index among regions for *A*. *digitifera* and *A. tenuis* (Nishikawa and Sakai 2005a) is not correlated with maximum pelagic larval duration until settlement (*A*. *digitifera*: 54 days, *F*_ST_ = 0.021, *P* < 0.01; *A. tenuis*: 69 days, *F*_ST_ = 0.048, *P* < 0.01). Also, local genetic differentiation (*F*_SC_ among populations = 0.039, *P* < 0.001) was detected among sites in *C. aspera*, which employs planula brooding as well as broadcast spawning (Nishikawa and Sakai 2005b). *Galaxea fascicularis* appears to maintain the genetic diversity and connectivity within types by the virtue of the broadcast spawning. On the other hand, asexual reproduction probably contributes to the population maintenance because multiple colonies are often distributed locally (Nishihira and Veron 1995). Furthermore, Nakajima et al. (2015) confirmed extensive clonality, although clonal diversity also has not been determined at other sites. We assessed genetic differentiation between mt‐L and mt‐S types in the Nansei Islands using cross‐type microsatellite markers developed by Nakajima et al. (2015). Furthermore, we analyzed clonal diversity, genetic diversity, and differentiation within types at four regions in the Nansei Islands. In addition, we searched for novel genetic clusters (other types) in this species because two types had been identified based only on tentacle characters and a mitochondrial DNA noncoding region.

## Materials and Methods

### Study areas and collection of coral specimens

Sampling sites were located around the Nansei Islands (17 sites in four regions; Fig. 1, Table 1). The maximum distance between sites is greater than 750 km, between Ayamaru at Amami Island (A‐Aya) and Amitori in the Yaeyama Islands (Y‐Ami). We randomly collected a single branch from each colony of *G. fascicularis*. Specimens were preserved in ethanol and brought to the laboratory. The dataset from Zampa is from Nakajima et al. (2015).

**Table 1:** Geographic location information and population genetic parameters for each site. *N*: the number of analyzed specimens. *G*: the number of multilocus genotypes (MLGs). *MLL*: the number of multilocus lineages (MLLs), considering somatic mutation and scoring error and including identical genotypes resulting from sexual reproduction by chance, estimated by *P*_SEX_ values for each site. *R* = (*MLL*−1)/(*N*−1). Because sampling strategies were not necessarily identical at all locations, clonal diversity cannot be strictly compared between sites; however, it is apparent that clonality varies between populations, based upon environmental factors, for example, wave action, which can cause increased fragmentation

Region	Location	Code	Latitude (N)	Longitude (E)	*N*_total_	mt‐L				mt‐S				mt‐L+			
						*N*	*G*	*MLL*	*R*	*N*	*G*	*MLL*	*R*	*N*	*G*	*MLL*	*R*
Amami	Ayamaru	A‐Aya	28°28′34″	129°43′00″	15	10	9	9	0.89	5	5	5	1.00	0	0	0	–
	Katetsu	A‐Kat	28°08′09″	129°20′39″	6	0	–	–	–	6	6	6	1.00	0	0	0	–
	Kuninao	A‐Kun	28°22′29″	129°24′14″	33	14	14	14	1.00	19	19	19	1.00	0	0	0	–
Okinawa	Zampa	O‐Zam	26°26′20″	127°42′40″	97	53	10	10	0.17	44	10	7	0.14	0	0	0	–
	Odo	O‐Odo	26°05′17″	127°42′27″	13	6	5	5	0.80	7	6	5	0.67	0	0	0	–
	Kume	O‐Kum	26°19′15″	126°51′24″	39	20	20	18	0.89	9	9	8	0.88	10	10	10	1.00
Miyako	Ikema	M‐Ike	24°56′02″	125°13′50″	14	9	9	9	1.00	5	5	5	1.00	0	0	0	–
	Yoshino	M‐Yos	24°44′52″	125°26′41″	27	24	2	1	0.00	3	2	2	0.50	0	0	0	–
	Ueno	M‐Uen	24°43′05″	125°20′30″	53	6	6	5	0.80	46	8	8	0.16	1	1	1	–
Yaeyama	Hirakubo	Y‐Hir	24°35′35″	124°18′29″	12	5	2	2	0.25	7	7	7	1.00	0	0	0	–
	Ohama	Y‐Oha	24°20′24″	124°11′55″	38	36	3	3	0.06	2	1	1	0.00	0	0	0	–
	Kannon	Y‐Kan	24°21′55″	124°06′40″	17	10	9	9	0.89	7	7	6	0.83	0	0	0	–
	Taketomi	Y‐Tak	24°20′40′	124°05′21″	30	14	10	9	0.62	16	14	14	0.87	0	0	0	–
	Kuroshima	Y‐Kur	24°18′05″	124°00′56″	13	4	4	4	1.00	9	9	8	0.88	0	0	0	–
	Nakano	Y‐Nak	24°25′52″	123°47′26″	37	35	10	7	0.18	2	2	2	1.00	0	0	0	–
	Amitori	Y‐Ami	24°19′47″	123°41′46″	39	37	27	16	0.42	2	2	2	1.00	0	0	0	–
	Haemida	Y‐Hae	24°16′06″	123°49′47″	27	12	7	3	0.18	15	15	15	1.00	0	0	0	–
Total					510	295	147	124		204	127	120		11	11	11	

### Scoring genotypes of microsatellites and identification of mitochondrial DNA sequence type

Genomic DNA was extracted using a DNeasy Blood & Tissue kit (Qiagen, Hilden, Germany). To analyze polymorphism and amplification of designed primer sets and to identify the mitochondrial DNA sequence type (mitochondrial type), we used the tailed primer method to perform PCR. We developed 11 microsatellite markers from *G. fascicularis* mt‐L type, which are available for both mt‐L and mt‐S types (Nakajima et al. 2015). Three of 11 loci frequently indicated complex genotypic patterns showing a 1‐bp shift among some alleles, complicating the process of determining genotypes. As a result, eight microsatellite markers were used for scoring genotypes in this study (see Table S1). The reaction mixture (5 μL) contained template DNA (<100 ng), AmpliTaq Gold 360 Master Mix (Thermo Fisher Scientific, Waltham, MA, USA), and three primers for each locus: a non‐tailed forward primer (0.2 μM), a reverse primer with a U19 sequence tail (0.2 μM), and a U19 (5′‐GGTTTTCCCAGTCACGACG‐3′) primer (0.5 μM) fluorescently labeled with FAM, VIC, or NED, based on the method of Schuelke (2000). Furthermore, 188‐1 (5′‐GAATAGGGTATACTAGCAGGTC‐3′, see Watanabe et al. 2005), 188‐R3‐U19 (5′‐GGTTTTCCCAGTCACGACGCATCATTATCCTCTTCAAGG‐3′), and U19 primers fluorescently labeled with VIC were used to identify the mitochondrial type (mt‐L: 460 bp or mt‐S: 170 bp) of the noncoding region between *cyt* b and *nad* 2. Amplifications of all microsatellite loci and the noncoding region between *cyt* b and *nad* 2 were performed with the following PCR conditions: 95°C for 9 min; followed by 35 cycles at 95°C for 30 s, 54°C for 30 s, and 72°C for 1 min; and a final extension at 72°C for 5 min. Amplified PCR products with the internal size standards GeneScan 600 LIZ (Thermo Fisher Scientific) were analyzed using an automated capillary‐based DNA sequencer (ABI 3130xl Genetic Analyzer, Thermo Fisher Scientific) and GeneMapper ver. 3.7 (Thermo Fisher Scientific).

To compare the sequence of the mitochondrial noncoding region between *cyt* b and *nad* 2 to the one reported previously, PCR products amplified using 188‐1 and 188‐R3 (5′‐CATCATTATCCTCTTCAAGG‐3′) for ten colonies (composed of mt‐L, mt‐S, and mt‐L+ collected in O‐Kum, see Results about mt‐L+) were purified using ExoSAP‐IT (Affymetrix, Santa Clara, CA, USA) and were sequenced bidirectionally using a BigDye Terminator Kit ver. 3.1 (Thermo Fisher Scientific). Products for sequencing were purified by ethanol precipitation, and sequences were analyzed using an ABI 3730xl Genetic Analyzer (Thermo Fisher Scientific). Colonies with both mitochondrial types by fragment analysis (see Results) were also sequenced to confirm the sequences of the mitochondrial noncoding region.

### Detection of clonal replicates

Multilocus lineages (MLLs) were employed to classify clonal replicates (Arnaud‐Haond et al. 2007a). Genotyped colonies from the same site, displaying differences at no more than one locus, were considered clones derived from fragmentation (asexual reproduction), to avoid the misidentification of multilocus genotypes by incorrect genotyping caused by somatic mutation or scoring error. When slightly different genotypes were determined to belong to the same MLL, the most common genotype was used to represent the MLL, following the method of Arnaud‐Haond et al. (2007b). However, if the most common genotype could not be determined (e.g., if two genotypes occurred in equal numbers), the genotype was set to zero so that this locus in the MLL was excluded from further analyses. MLLs were counted to estimate clonality using GenAlEx ver. 6.5 (Peakall and Smouse 2006).

We also calculated the probability of a given multilocus genotype (MLG) occurring *n* times within a given type and site, repeated as a consequence of different recombination events (*P*_SEX_ was calculated considering the *F*_IS_ estimates in the dataset) using GenClone ver. 2.0 (Arnaud‐Haond and Belkhir 2007). We removed one or two loci for the calculation of exact *P*_SEX_ values if a somatic mutation or a scoring error appeared in the target MLL. We retained the replicated MLG in an MLL if two or more MLGs were the same, but occurred by chance as a result of sexual reproduction. However, if a population contained only one or several genotypes, even with a very high sampling effort, and no further MLGs were detected, the statistical power associated with *P*_SEX_ could be low (Arnaud‐Haond et al. 2007b). In such a case, we did not consider the *P*_SEX_ value to accurately estimate the replicated MLG and assumed that clonal reproduction accounted for such similarities.

Clonal diversity was estimated with the following index proposed by Dorken and Eckert (2001): *R* = (*MLL*−1)/(*N*−1) (*MLL*: the number of multilocus lineages, *N*: the number of colonies analyzed). We removed clonal replicates from the dataset, and MLLs were retained for the following population genetic analyses.

### Statistical analyses for population genetic indexes

The number of alleles, values of observed and expected heterozygosity (*H*_O_ and *H*_E_, respectively), and a deviation index (*F*_IS_) from Hardy–Weinberg equilibrium (HWE) for each type were evaluated with GenAlEx for each region. INEST ver. 2.0 (Chybicki and Burczyk 2009) was used to estimate the null allele frequency and the extent of inbreeding in each region. Using a Bayesian approach (individual inbreeding model), INEST was run using nfb (null alleles, inbreeding coefficients, and genotyping failures) and nb (null alleles and genotyping failures) models to detect the existence of inbreeding effects in our dataset with 50,000 burn‐in cycles and 500,000 cycles overall. Postprocessing was conducted to calculate the mean null allele frequency of eight microsatellite loci, the mean value of the inbreeding coefficient, and the limit of the highest density posterior interval. The deviance information criterion (DIC) was used to determine the best model. For the estimation of genetic diversity, we calculated allelic richness (*A*_R_) at each region using FSTAT ver. 1.2 (Goudet 1995). Furthermore, the *H*_E_ value was also used as the index of genetic diversity. Genetic differentiation was estimated by a hierarchical analysis of molecular variance (AMOVA; Excoffier et al. 1992) using GenAlEx. The genetic differentiation index between regions, pairwise *F*_ST_, was also calculated using GenAlEx. The significance of each *F*_ST_ value was tested with 999 permutations. To estimate the effect of null alleles to the *F*_ST_ values, we calculated *F*_ST_ with both including null alleles (INA) and excluding null alleles (ENA) effects using FreeNA (Chapuis and Estoup 2007; Chapuis et al. 2008). The number of replicates was 1000 for the computation of the bootstrap 95% confidence intervals.

The population structure for all MLLs based on Bayesian clustering was inferred using STRUCTURE ver. 2.3.4 (Pritchard et al. 2000). We ran STRUCTURE on the data for 10 independent chains for each *K* value (*K* = 1 to 11). A burn‐in of 100,000 iterations followed by 1,000,000 Markov chain Monte Carlo (MCMC) replications was used for population clustering without prior information under the admixture model and assuming correlated allele frequencies (Falush et al. 2003). In the admixture model, individuals were assumed to have drawn exclusively from the *K* genetic clusters and were allowed to have a mixed ancestry (Pritchard et al. 2000; Falush et al. 2003). After calculating the mean log probability, Ln P(D), the number of clusters (*K*) that best fit the data, was determined using the method of Evanno et al. (2005), as implemented in STRUCTURE HARVESTER (Earl and vonHoldt 2012). ∆*K* is an ad hoc quantity for predicting the number of possible clusters (Evanno et al. 2005). These data were also merged and outputted using CLUMPAK (Kopelman et al. 2015). To infer the population genetic structure within type, another Bayesian clustering algorithm was implemented in InStruct (Gao et al. 2007), which is an extension of the approach of STRUCTURE for simultaneous inference of inbreeding or selfing rates and population‐of‐origin classification. This procedure was used because inbreeding was suggested as a possible reason for the high inbreeding coefficients in our dataset (see Results). This algorithm analyzed and eliminated HWE assumptions within possible genetic clusters and calculated the expected genotype frequencies based on inbreeding or selfing rates (Gao et al. 2007). We ran InStruct for 10 independent chains for each *K* value (*K* = 1 to 5) and type. Each chain was iterated 1,000,000 times including burn‐in with 100,000 iterations. Determination of the number of *K* clusters that best fit the data was conducted using the method of Evanno et al. (2005). These data were merged and outputted using CLUMPP ver. 1.1.2 (Jakobsson and Rosenberg 2007) and distruct ver. 1.1 (Rosenberg 2004), respectively.

## Results

### Determination of mitochondrial type

**Figure 2:**
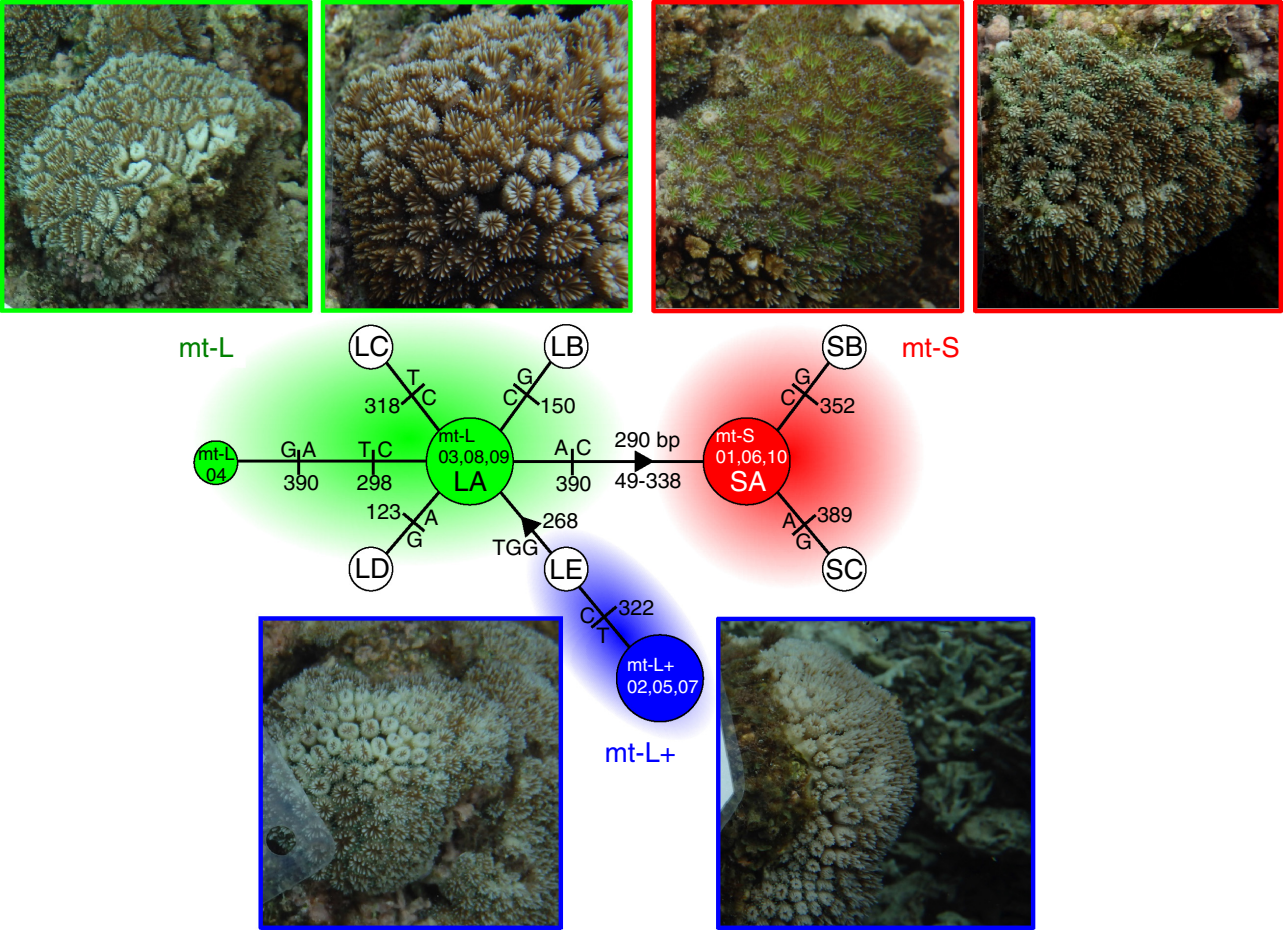
Mitochondrial DNA sequences revealed a previously unknown type of *Galaxea fascicularis*. A haplotype network comparing the three types obtained from 10 specimens from O‐Kum in Okinawa with those defined in previous studies, based upon specimens collected in the Ryukyu Archipelago (Watanabe et al. 2005). The three types, readily identified by mitochondrial DNA, cannot be reliably distinguished based on appearance. Sequences of LA to LE and sequences of SA to SC were obtained from Watanabe et al. (2005). The sequence of LE was genetically closest to type mt‐L+ from this study. Colors correspond to the clusters in the Bayesian clustering analysis (*K* = 3, see Fig. 3A).

Of 531 colonies of *G. fascicularis* collected from 17 sites at four regions in the Nansei Islands, we successfully determined the multilocus microsatellite genotypes and mitochondrial type in all but 21 colonies. When both peaks were indicated from the mitochondrial noncoding region between *cyt* b and *nad* 2, the main peak was used to decide the mitochondrial type if the other peak was extremely small (less than one‐tenth the height of the main peak). Fourteen of these 21 are indeterminate colonies, and distinct and clear fragment peaks for both mitochondrial types were detected, although only one peak is usually derived from the mitochondrial noncoding region between *cyt* b and *nad* 2. Both peaks were derived from both types of mitochondrial noncoding region by sequencing. We excluded these 21 colonies and used genotypes from the remaining 510 colonies for all other analyses. Detailed site data are provided in Table 1. Of 510 colonies, 295 were identified as mt‐L, while 204 were mt‐S. Both mt‐L and mt‐S were found at 16 sites, excluding A‐Kat (Table 1). In ten colonies collected at O‐Kum and one at M‐Uen, we found an unexpected mitochondrial type that showed 3 bp (TGG) more than the usual mt‐L fragment length from the noncoding region. We named the unexpected type mt‐L+. Haplotype networks from sequencing the mitochondrial noncoding region are shown with the sequence output in the study by Watanabe et al. (2005) (Fig. 2). As a result, this mt‐L+ type is similar to the mt‐L type E lineage collected at st.6 in Yaeyama in the previous study.

### Multilocus lineages and inbreeding coefficients

**Figure 3:**
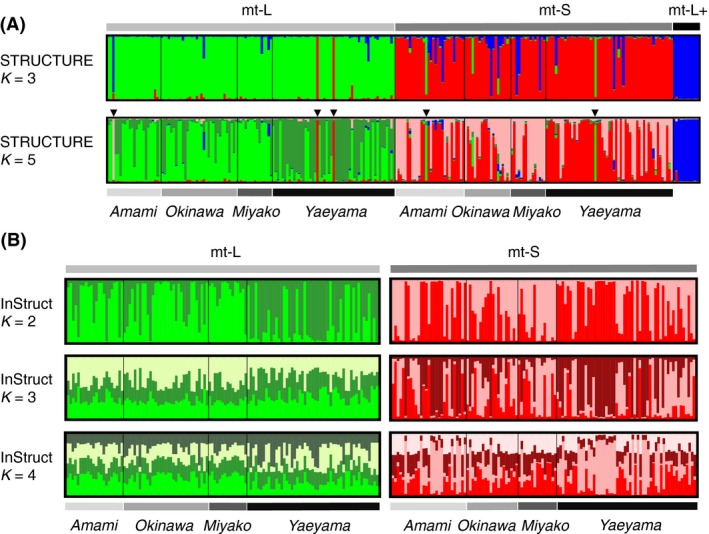
Mitochondrial DNA sequence types are genetically well differentiated. (A) Most multilocus lineages (MLLs) were correctly assigned by Bayesian clustering analysis when the number of clusters was set to 5. These clusters corresponded closely to the three *G. fascicularis* types. The *X*‐axis shows the probability of MLL membership in each cluster. Using STRUCTURE with the number of groups set to five (*K* = 5), three MLLs in mt‐L and two MLLs in mt‐S showed a reversed genetic cluster (see black triangles). (B) Finer genetic differentiation was detected in each type among regions using InStruct instead of STRUCTURE, which is sensitive to high inbreeding coefficients (see Table 2). Using microsatellite markers, STRUCTURE classified five MLLs differently than their mitochondrial DNA sequence types. These MLLs were excluded from analyses with InStruct.

Multilocus genotypes were determined from 295 mt‐L, 11 mt‐L+, and 204 mt‐S colonies. Eight microsatellite loci utilized for scoring genotypes displayed 124, 11, and 120 MLLs, respectively (Table 1). The same MLG occurred by chance as a result of sexual reproduction in an mt‐S MLL at M‐Uen, based upon the *P*_SEX_ value. We added this doubled MLL as a different lineage for subsequent analyses. In these MLLs, there is a large variation in clonality at each site. Values ranged from 0 to 1 for both mt‐L and mt‐S types, although MLLs were few at some sites because of high clonal diversity within types. Three MLLs (corresponding to three colonies analyzed) in mt‐L and two MLLs (corresponding to two colonies analyzed) in mt‐S showed the opposite genetic cluster in STRUCTURE (see black triangles in Fig. 3A). These five MLLs were excluded for InStruct and further statistical analyses to avoid misleading genotypic data.

Our dataset displayed high *F*_IS_ values (*F*_IS_ = 0.122 to 0.256 for each region), and this result was supported by a relatively high frequency of null alleles (Null freq. = 0.061 to 0.140 among regions) and inbreeding influences (Avg Fi = 0.021 to 0.133 among regions) from the results of INEST (Table 2). Of nine populations including mt‐L+, the nfb model is the best in two populations and nb is the best in seven populations; however, the difference in DIC values between models is <1.0 in seven populations, except for mt‐L in Miyako and mt‐L+.

**Table 2:** *A*_R_: allelic richness, the standardized mean allele number for eight microsatellite loci. *H*_O_ and *H*_E_ are observed and expected heterozygotes, respectively. *F*_IS_: deviation index from Hardy–Weinberg equilibrium. Null allele frequency (Null freq.), inbreeding coefficient (Avg Fi), and the 95% highest posterior density intervals for each region using a Bayesian approach. INEST was run using nfb (null alleles, inbreeding coefficients, and genotyping failures) and nb (null alleles and genotyping failures) models to detect inbreeding effects. The deviance information criterion (DIC) was used to determine the best model (shown in bold)

Type	Region	*A*_R_	*H*_O_	*H*_E_	*F*_IS_	Null freq.	Avg Fi (95%CI)	DIC (nfb)	DIC (nb)
mt‐L	Amami	11.36	0.751	0.899	0.163	0.080	0.033 (0–0.100)	1729.842	**1729.312**
	Okinawa	11.94	0.772	0.907	0.150	0.061	0.051 (0–0.124)	**2775.180**	2775.290
	Miyako	10.57	0.724	0.886	0.187	0.085	0.031 (0–0.106)	1109.643	**1108.173**
	Yaeyama	11.65	0.727	0.917	0.209	0.116	0.021 (0–0.062)	4183.421	**4182.616**
mt‐S	Amami	11.03	0.748	0.889	0.157	0.090	0.020 (0–0.060)	2215.322	**2214.402**
	Okinawa	11.14	0.712	0.872	0.179	0.104	0.035 (0–0.107)	1492.341	**1492.242**
	Miyako	10.14	0.654	0.843	0.223	0.072	0.133 (0–0.266)	**1032.335**	1033.250
	Yaeyama	10.26	0.644	0.870	0.256	0.140	0.024 (0–0.069)	3843.593	**3842.695**
mt‐L+		7.95	0.545	0.621	0.122	0.064	0.026 (0–0.092)	554.795	**552.780**

### Genetic differentiation among mitochondrial types

Significant genetic differentiation among types was detected based on AMOVA (*F*_RT_ = 0.054, *P* < 0.001, Table 3). The genetic differentiation between mt‐L and mt‐S was significant in all cases (pairwise *F*_ST_ = 0.034 to 0.068, all *P*s = 0.001, Table 4). Genetic differentiation between mt‐L+ and other types was also significant, and these values are higher than values between mt‐L and mt‐S (between mt‐L and mt‐L+: pairwise *F*_ST_ = 0.158 to 0.179, all *P*s = 0.001; between mt‐S and mt‐L+: pairwise *F*_ST_ = 0.158 to 0.183, all *P*s = 0.001). These tendencies are identical with the results of pairwise *F*_ST_ excluding the effect of null alleles, and no large influence of null alleles for the calculation of these genetic differentiation indexes (Table S2). The results from STRUCTURE, based on Bayesian statistical model‐based clustering, also indicated that these three types are genetically isolated (Fig. 3A). Two peaks are observed in the graph of ∆*K* value, which is an index to estimate the greatest possible number of assumed clusters (∆*K* = 187.96 at *K* = 3 and ∆*K* = 48.49 at *K* = 5, Fig. S1). This application of the method of Evanno et al. (2005) indicated that *K* = 3 offers the best explanation for the genetic data.

**Table 3:** The three mitochondrial DNA sequence types are genetically well differentiated, showing low variation within types. Analysis of molecular variance (AMOVA), showing degrees of freedom (df), sum of squares (SS), variance components (Var.), percentage of variances (%), and *F*‐statistics between types, among regions within types and within regions

Source	df	SS	Var. (%)	*F*‐statistics (*P* value)
Among types	2	69.331	0.209 (5.4%)	*F*_RT_ = 0.054 (0.001)
Among regions within type	6	40.454	0.057 (1.5%)	*F*_SR_ = 0.016 (0.001)
Within regions	491	1758.089	3.581 (93.1%)	*F*_ST_ = 0.069 (0.001)
Total	499	1867.874	3.847 (100.0%)	

**Table 4:** Pairwise *F*_ST_ values for all mitochondrial DNA sequence types as an index of genetic differentiation between pairs of regions. The lower left table shows pairwise *F*_ST_ values, and the upper right shows *P* values. Larger *F*_ST_ values denote greater genetic differentiation. Because the number of mt‐L+ specimens was small (*MLL* = 11), all specimens are combined into a single population

Type	Region	mt‐L				mt‐S				mt‐L+
		Amami	Okinawa	Miyako	Yaeyama	Amami	Okinawa	Miyako	Yaeyama	
mt‐L	Amami		0.005	0.001	0.001	0.001	0.001	0.001	0.001	0.001
	Okinawa	0.009		0.001	0.002	0.001	0.001	0.001	0.001	0.001
	Miyako	0.024	0.013		0.002	0.001	0.001	0.001	0.001	0.001
	Yaeyama	0.016	0.009	0.019		0.001	0.001	0.001	0.001	0.001
mt‐S	Amami	0.037	0.034	0.046	0.039		0.010	0.001	0.001	0.001
	Okinawa	0.037	0.042	0.053	0.043	0.009		0.003	0.001	0.001
	Miyako	0.050	0.052	0.054	0.053	0.021	0.017		0.001	0.001
	Yaeyama	0.058	0.057	0.068	0.055	0.014	0.014	0.032		0.001
mt‐L+		0.179	0.165	0.171	0.158	0.158	0.173	0.183	0.178	

### Genetic diversity and differentiation within mitochondrial types

Allelic richness and the expected heterozygosity of mt‐L and mt‐S at northern regions were comparable to those of southern regions (Table 2). Allelic richness values ranged from 10.57 to 11.94 in mt‐L and from 10.14 to 11.14 in mt‐S. In mt‐L+ collected at O‐Kum and M‐Uen, the value was 7.95. For the expected heterozygosity, values ranged from 0.886 to 0.917 in mt‐L and from 0.843 to 0.889 in mt‐S. The value of mt‐L+ collected at O‐Kum was 0.621. Compared with the results of genetic differentiation among types, genetic differentiation within type (mt‐L or mt‐S, but not mt‐L+) showed significant variation among regions. Values ranged from 0.009 to 0.024 in mt‐L and from 0.009 to 0.032 in mt‐S (Table 4). The extent of genetic differentiation was similar in both mt‐L and mt‐S. These tendencies are also identical with the results of pairwise *F*_ST_ excluding the effect of null alleles (Table S2). STRUCTURE results (Fig. 3B) suggest slight genetic structuring among regions of each type. A slight genetic discontinuity occurs between Miyako and Yaeyama in mt‐L. On the other hand, a slight genetic discontinuity is also observed between Miyako and Yaeyama in mt‐S, resembling that cluster composition is found in Amami and Yaeyama. The results of InStruct, considering inbreeding coefficients, also showed the same tendency. A peak was observed in the graph of the ∆*K* value at *K* = 2 in both mt‐L and mt‐S (∆*K* = 311.28 in mt‐L and 729.49 in mt‐S, Fig. S2).

## Discussion

For the broadcast‐spawning coral, *G. fascicularis*, in the Nansei Islands of Japan, this study analyzed mitochondrial types and compared these types with genotypes of microsatellites to estimate the species diversity of *Galaxea*. Mitochondrial type is related to microsatellite genotype in all regions. Values of clonal diversity are variable among sites for each type. Genetic differentiation is significant, but low among regions, and genetic diversity has been maintained for each type.

### Genetic differentiation among mitochondrial types

Although mt‐L colonies were more numerous than mt‐S colonies, the ratio of types varies widely among the sites that we sampled. This pattern agrees with the previous studies (Watanabe et al. 2005; Abe et al. 2008a). All sites, excluding A‐Kat, possessed both types, and the ratio is unrelated to latitude in the Nansei Islands. Colonization may be more influenced by evolutionary processes than by latitudinal factors such as temperature. Evolutionary divergence of tropical coral species is likely to occur in the center of the tropical region, known as the Coral Triangle. However, the genus *Galaxea* has an extensive range in the Indo‐western Pacific, and it is not clear what sort of divergence has occurred in the Coral Triangle itself. In Okinawa, mt‐L and mt‐S types can be distinguished not only by morphology, but also by the timing of spawning (Watanabe et al. 2005); mt‐L spawns a few weeks earlier than mt‐S in some cases. With this slight but significant genetic differentiation between mt‐L and mt‐S, segregated spawning may explain genetic differentiation between types. However, the spawning season of mt‐L+ is not known. Further observation will be needed to understand the reproductive strategies contributing to genetic isolation among types in this species.

The mitochondrial type, mt‐L+, found at O‐Kum and M‐Uen, was unexpected, indicating greater genetic differentiation; however, the sequence of the mitochondrial noncoding region is interestingly similar to the E lineage of mt‐L reported by Watanabe et al. (2005) (Fig. 2). Although *G. fascicularis* is easily distinguished from other *Galaxea* species by morphology, we cannot exclude the possibility that this unexpected type is a known *Galaxea* species that has previously been reported in the Nansei Islands, such as *Galaxea astreata* or *Galaxea pauciradiata* (formerly *Galaxea paucisepta*) (see Nishihira and Veron 1995; Veron 2000). However, we could not differentiate morphological types in the field (see Fig. 2). Morphological characterization of *Galaxea* appears to be more difficult than we anticipated, as in some other corals. For example, the genus *Pocillopora* also shows interspecific ambiguity with high levels of morphological variability in colony shape and branch size, but these well‐known variants do not appear to correlate with genetic variation (Pinzón and LaJeunesse 2011). Nevertheless, the morphs are distinguished by microsatellite markers and mitochondrial DNA regions (ORF and control region) (Pinzón and LaJeunesse 2011; Pinzón et al. 2013; Schmidt‐Roach et al. 2013, 2014). Thus, genetic identification has contributed to species classification and estimates of species diversity of corals. We tried to compare differences between *G. fascicularis* and other *Galaxea* species using gene sequences from GenBank. We found six gene sequences commonly shared by *G. fascicularis* and *G. astreata*. However, large clear differences between types cannot be confirmed using the haplotype network by TCS ver. 1.21 (Clement et al. 2000). Some registered sequences may actually be derived from other genera because some of the haplotypes from the same nominal species are separated (Fig. S3). Understanding the phylogenetic relationship of *Galaxea*, more phylogenetic analyses (e.g., genome‐wide single nuclear polymorphism (SNP) markers, or amino acid sequences derived from all coding genes on mitochondrial genome) are needed for the exact estimation of species diversity. With nematocyst types, fine‐scale investigations of the corallite and subcorallite features observed with scanning electron microscopy (SEM) and thin sections may reveal defining characters each type.

### Interbreeding and ambiguous mitochondrial type

Intermediate genetic lineages were also detected with STRUCTURE. Interbreeding is less likely to occur between the two types than within types, suggesting that spawning seasons do not overlap in some cases (Watanabe et al. 2005). Abe et al. (2008b) reported that the seasons are not completely separated because they managed to conduct fertilization experiments between types. Therefore, a reproductive isolation may not be complete between mt‐L and mt‐S, which may have resulted from a recent evolutionary speciation process. Although cross‐type hybrids occurred as a result of fertilization experiments between types (Abe et al. 2008b), the incubation time (4–5 h) with higher sperm concentration appears to be longer than those that occur in nature (see Iguchi et al. 2009; Nozawa et al. 2015). Therefore, as Watanabe et al. (2005) suggested, actual hybridization between types may be rare, even if spawning seasons partly overlap in some colonies. Different spawning times appear to maintain the genetic divergence between types. Two sympatric *Acropora* species, *A. digitifera* and a cryptic species *Acropora* sp. 1 aff. *digitifera* (Hayashibara and Shimoike 2002), also show significant genetic differentiation (Nakajima et al. 2012b). The octocoral species, *Heliopora coerulea*, is also separated into two genetic clusters that are related to branch morphotype (Yasuda et al. 2014). Compared with *G. fascicularis*, these *Acropora* and *Heliopora* species are influenced by the difference in temporal reproductive isolation (*Acropora*: Hayashibara and Shimoike 2002; Ohki et al. 2015; *Heliopora*: Villanueva 2015).

We confirmed the existence of some colonies with both mt‐L and mt‐S peaks in the fragment analysis, although we excluded MLLs with undefined types from our analyses (see Results). Both mitochondrial types were confirmed in these colonies from the sequencing results. Although we suspected possible contamination in these specimens, repeated experiments did not reveal experimental errors. Furthermore, among microsatellite genotypes, there is no evidence of chimeric or triploid colonies, which are occasionally detected in genetic experiments (e.g., Baums et al. 2005; Puill‐Stephan et al. 2012). We believe that mitochondrial types in a colony may be derived from heteroplasmy by introgression of both parental mitochondria via hybridization between types in previous generations. Further organelle genome studies of corals may eventually resolve this question.

### Population maintenance of *Galaxea* within mitochondrial type

Species that comprise the frameworks of ecosystems, such as corals and seagrasses, often reproduce clonally (Baums et al. 2006). Small‐scale disturbances, such as waves during local storms, may increase the rates of clonal reproduction in coral populations as a result of fragmentation (Baums et al. 2006; Aranceta‐Garza et al. 2012). In *G. fascicularis,* both sexual and asexual reproductions contribute to population persistence and recovery from anthropogenic disturbances, showing a high degree of clonal diversity. Although we employed nonstandardized sampling among sites and did not standardize the sampling strategy due to the magnitude of the study area, spatial genetic and clonal structure within individual reefs is also informative (see Gorospe and Karl 2013). Further collection and analyses of specimens standardized among sites will provide the detailed clonal structure among populations, and spatial correlation based on locality data should elucidate kinship among colonies on a regional scale.

It is generally accepted that broadcast‐spawning corals have longer larval durations and higher connectivity among regions than brooding species. We expected that within types, *G. fascicularis* would also show high connectivity among regions and reduced genetic differentiation. Populations in high‐latitude areas and islands isolated from the central habitat of the species show decreased genetic diversity, and some other coral species reflect this tendency (e.g., Ayre and Hughes 2004; Ridgway et al. 2008; Davies et al. 2015). However, high genetic diversity was shown in high‐latitude peripheral populations of *Acropora* species (Nakajima et al. 2010; Noreen et al. 2013). Some *Acropora* species, classified as long‐term winner species, which increase in their relative contribution to the total habitat cover in survey site at Okinawa, are also pioneers following local extinctions due to disturbances such as mass bleaching events (van Woesik et al. 2011).

Two possible reasons can explain the significant genetic differentiation among regions within type. First, the larval duration may be shorter than for broadcast‐spawning corals, such as *Acropora*. Although larval duration is not certain for *G. fascicularis*, the first larval stage lasts only ~18 h before larvae start swimming (Okubo et al. 2013). Embryonic development of *G. fascicularis* is significantly faster than in *Acropora muricata* (Keshavmurthy et al. 2012). Second, we can explain high clonal diversity at some sites. Populations of *G. fascicularis* are largely maintained by fragmentation, which promotes the deviation from HWE by preventing random mating. In Bayesian clustering, slight genetic discontinuity occurs between Miyako and Yaeyama in mt‐L. On the other hand, it also exists between Miyako and Yaeyama in mt‐S, although similar cluster composition is found in Amami and Yaeyama. The difference between these two types may have resulted from physiological characteristics and historical transitions. Differences between types should be resolved in future studies to clarify the population dynamics and evolutionary history of each type.

In the Nansei Islands, this species maintains high genetic diversity and connectivity, as do other broadcast‐spawning coral species, such as *A. digitifera* (Nakajima et al. 2010). In the Nansei Islands, longer larval duration of the broadcast‐spawning corals probably contributes to high genetic diversity and connectivity, which may be enhanced by the Kuroshio Current and its branches. *Galaxea fascicularis* is also distributed in nonreef areas in the temperate zone of Japan (Nishihira and Veron 1995), but population sizes are small because that environment is harsh for tropical species. If the sea surface temperature gradually increases due to climate change, northern populations of this species may increase and its range may extend northward. As a result, other reef‐associated fauna also may extend their ranges as well.

## Conclusions

This population genetic study using microsatellite markers revealed different ratios of mt‐L and mt‐S types and clonal diversity among regions in *G. fascicularis*. Clonal diversity is related to geographic factors rather than to type or latitude. Further study may provide a picture of clonal structure within single reefs. We also defined genetic differentiation among types using multiple analyses. An unexpected mitochondrial type was found, and interestingly, this third type is genetically quite different from both the mt‐L and mt‐S types. The sequence of the mitochondrial noncoding region in this type is similar to that of a colony reported by Watanabe et al. (2005). This unexpected type is possibly a novel *Galaxea* species, although we cannot exclude the possibility that this type is a known *Galaxea* species with an ambiguous morphotype. Genetic differentiation among regions within types is lower, but still significant. With oceanographic factors as the Kuroshio Current and branch currents, both larval dispersal via sexual reproduction and fragmentation via asexual reproduction as biological characters appear to contribute to the maintenance of *Galaxea* populations in the Nansei Islands.

## Conflict of Interest

None declared.

## Supporting information


**Figure S1.** The optimal number of genetic clusters is three, using STRUCTURE from STRUCTURE HARVESTER. These three clusters correspond very closely to the three mitochondrial DNA sequence types identified from noncoding mitochondrial DNA sequences (mt‐L, mt‐S, mt‐L+). Mean Ln P(D) values (*K* = 1 to 11) across 10 iterations per *K*, and Δ*K* values (*K* = 2 to 10) using the method of Evanno et al. (2005).

**Figure S2.** The most likely number of genetic clusters is two for both mt‐L (a) and mt‐S (b) by estimation of the optimal number of genetic clusters of InStruct. Mean Ln P(D) values (*K* = 1 to 5) across 10 iterations per *K*, and Δ*K* values (*K* = 2 to 4) using the method of Evanno et al. (2005).

**Figure S3.** Haplotype networks based upon sequences of mitochondrial genes (12S rRNA, 16S rRNA, *atp* 6, *cox* 1, *cyt* b) and a nuclear gene (28S rRNA) from GenBank split nominal species, suggesting that some *Galaxea* species may either be misidentified or may contain cryptic species. These networks were constructed by TCS ver. 1.21 (Clement et al. 2000). Sequences of these six loci are registered in both *Galaxea fascicularis* and *Galaxea astreata*.

**Table S1.** Characteristics of eight polymorphic microsatellite loci in this study: locus name, repeat motif, primer sequence, size range of amplification products including U19 sequence, and GenBank accession number. U19, 5′‐GGTTTTCCCAGTCACGACG‐3′.

**Table S2.** Pairwise *F*_ST_ values calculated using FreeNA (Chapuis and Estoup 2007; Chapuis et al. 2008) to estimate the effect of null alleles. The values above the diagonal are pairwise *F*_ST_ by ENA (i.e., excluding null alleles) model and lower diagonal are by INA (i.e., including null alleles) model.
