# Supplementary figures and images for: Genetic differentiation and connectivity of morphological types of the broadcast‐spawning coral Galaxea fascicularis in the Nansei Islands, Japan
Source: Ecol Evol. 2016 Feb 3;6(5):1457–69. doi: 10.1002/ece3.1981 (PMC4775516; doi:10.1002/ece3.1981)

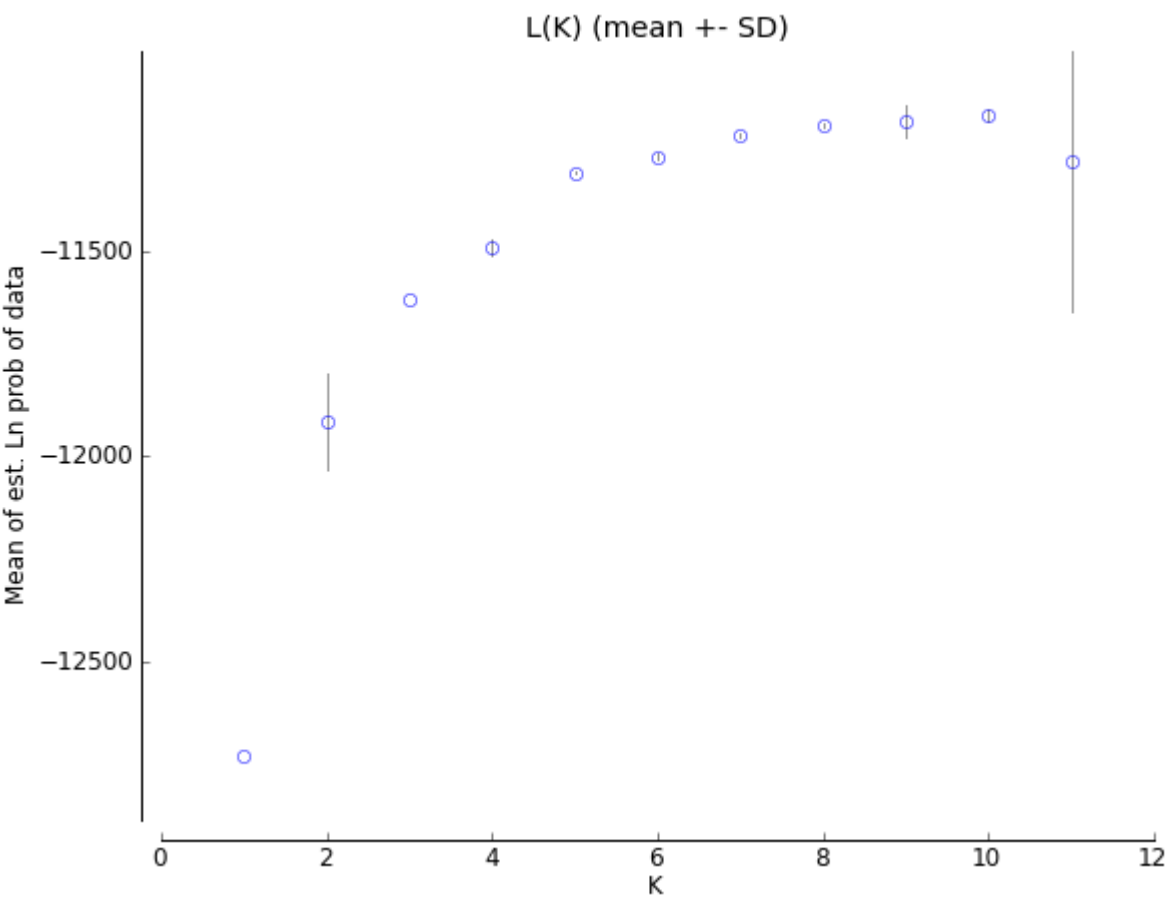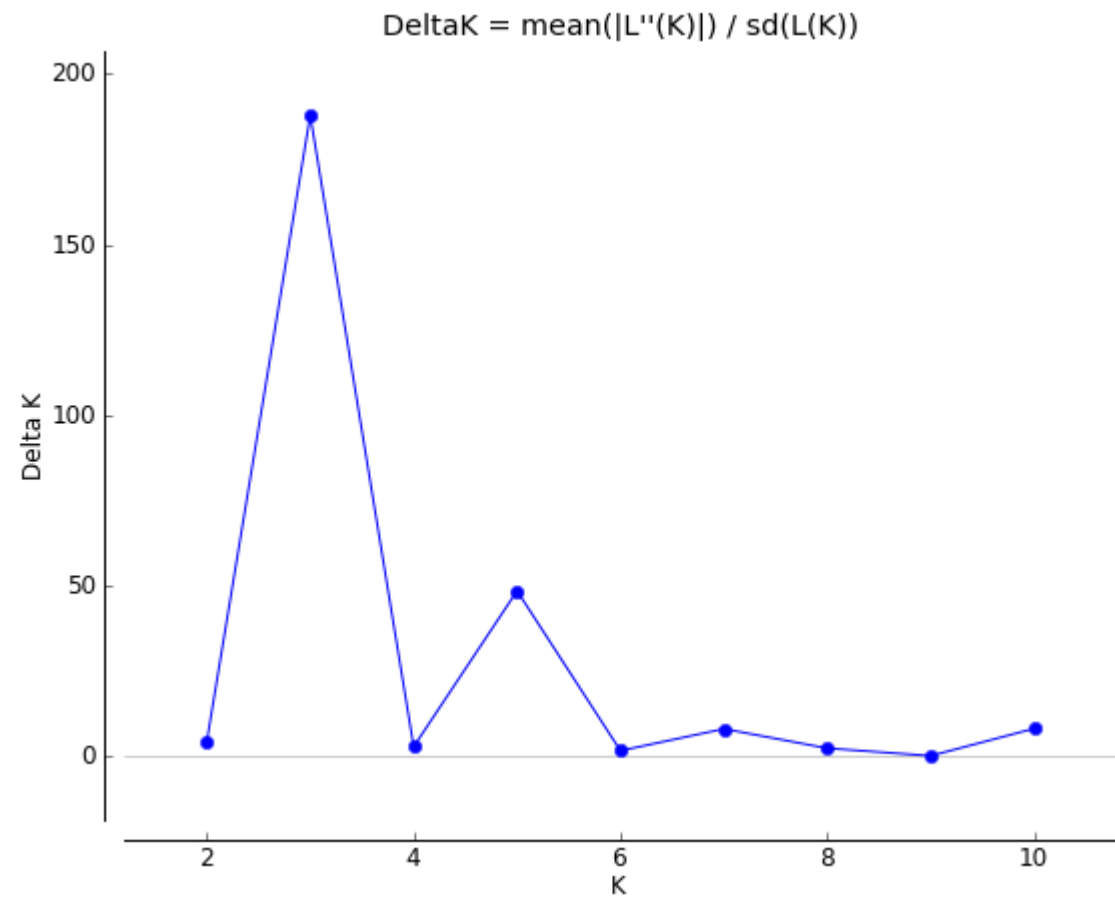

Fig.S1

Supplement: Supplementary file 1 — Figure S1. The optimal number of genetic clusters is three, using STRUCTURE from STRUCTURE HARVESTER. These three clusters correspond very closely to the three mitochondrial DNA sequence types identified from noncoding mitochondrial DNA sequences (mt‐L, mt‐S, mt‐L+). Mean Ln P(D) values (K = 1 to 11) across 10 iterations per K, and ΔK values (K = 2 to 10) using the method of Evanno et al. (2005). [file ECE3-6-1457-s001.pdf]

(A)

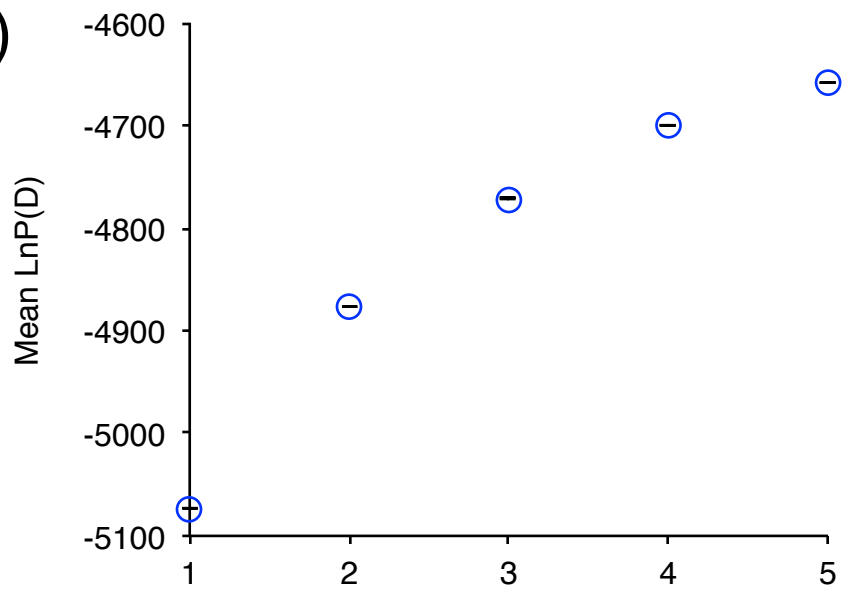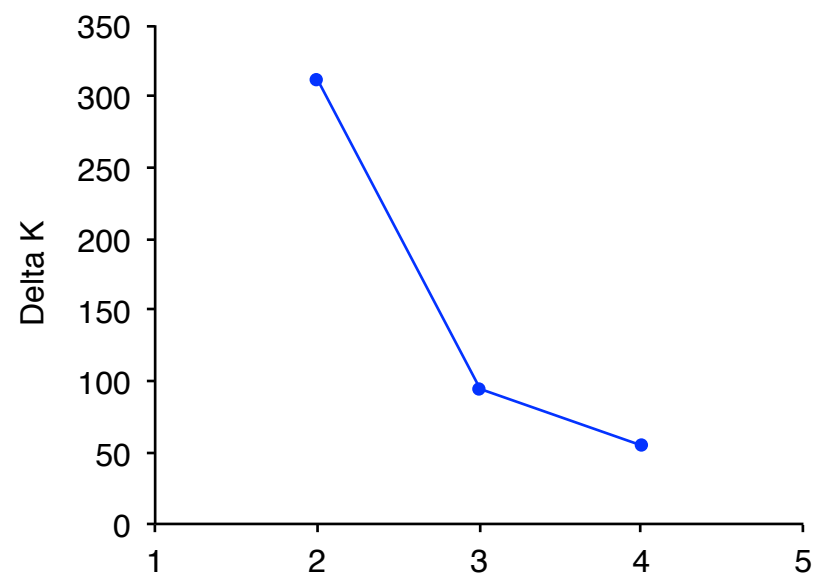

(B)

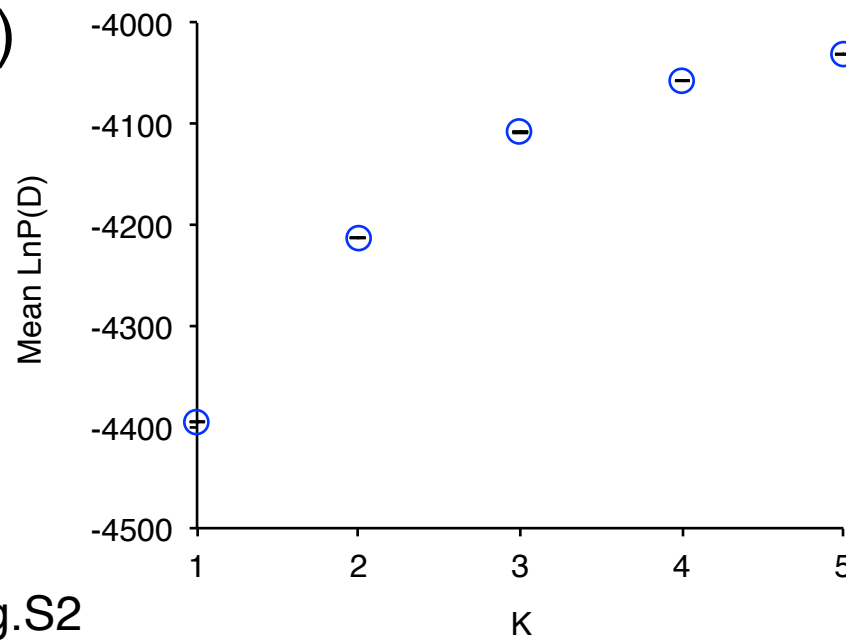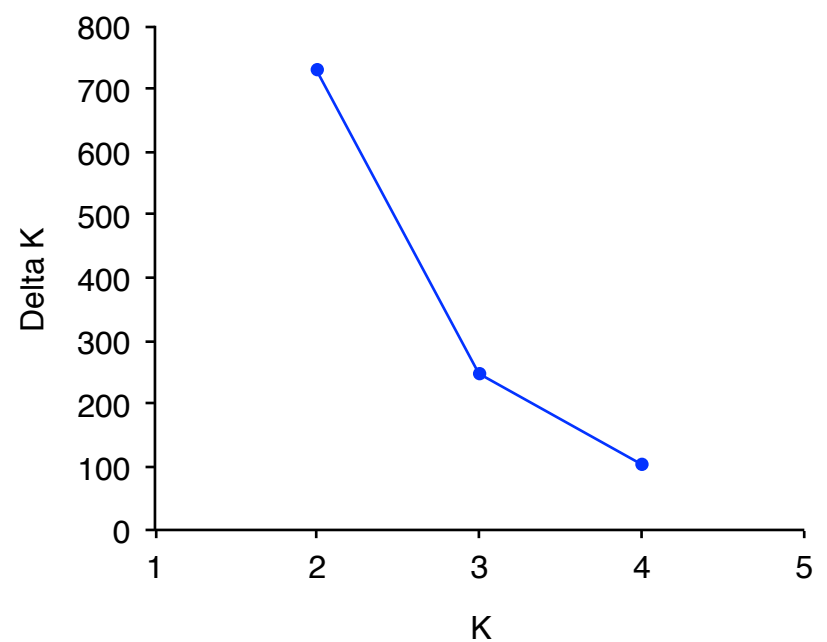

Fig.S2

Supplement: Supplementary file 2 — Figure S2. The most likely number of genetic clusters is two for both mt‐L (a) and mt‐S (b) by estimation of the optimal number of genetic clusters of InStruct. Mean Ln P(D) values (K = 1 to 5) across 10 iterations per K, and ΔK values (K = 2 to 4) using the method of Evanno et al. (2005). [file ECE3-6-1457-s002.pdf]

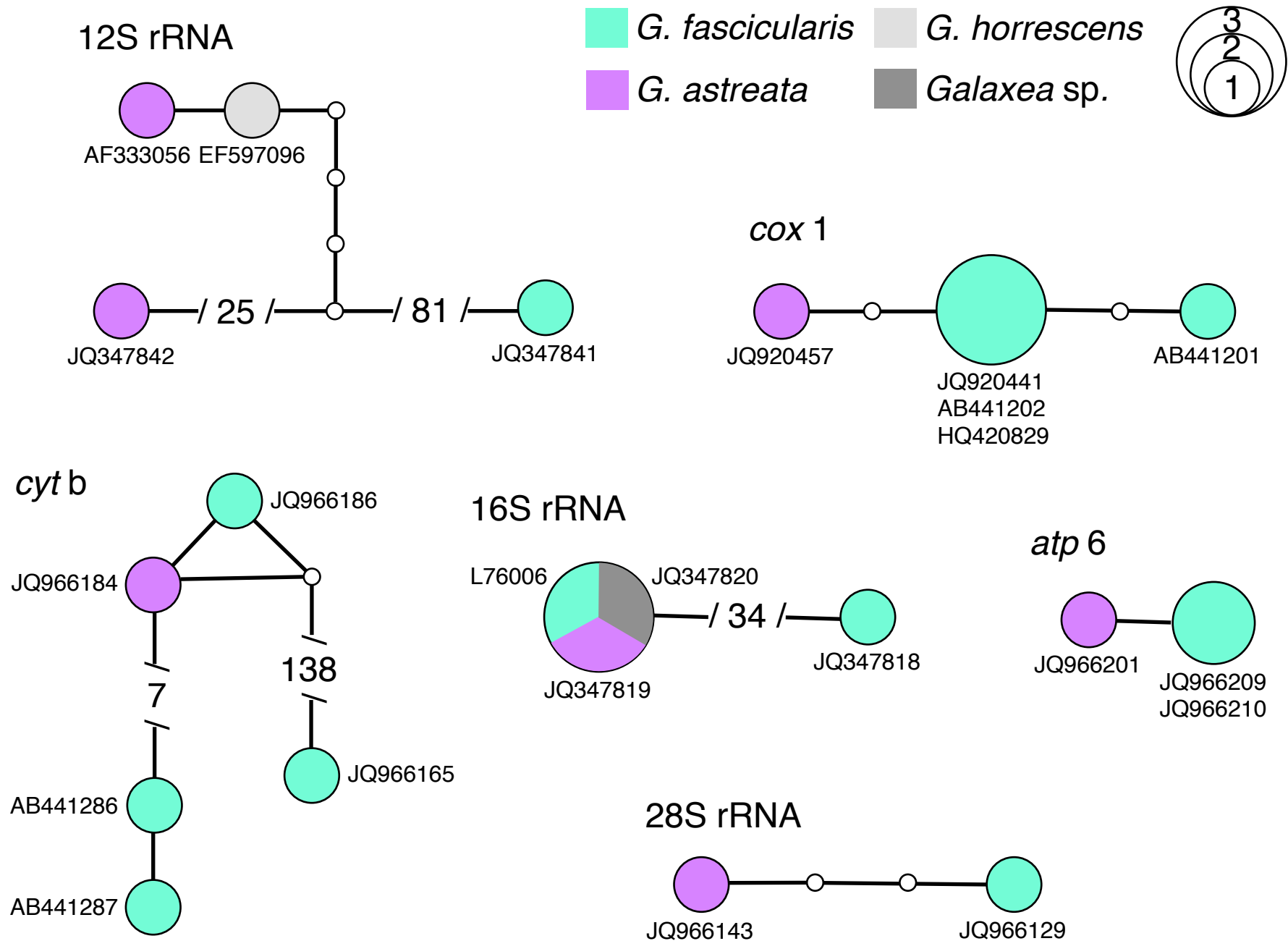

Fig.S3

Supplement: Supplementary file 3 — Figure S3. Haplotype networks based upon sequences of mitochondrial genes (12S rRNA, 16S rRNA, atp 6, cox 1, cyt b) and a nuclear gene (28S rRNA) from GenBank split nominal species, suggesting that some Galaxea species may either be misidentified or may contain cryptic species. These networks were constructed by TCS ver. 1.21 (Clement et al. 2000). Sequences of these six loci are registered in both Galaxea fascicularis and Galaxea astreata. [file ECE3-6-1457-s003.pdf]
